# Pre-pregnancy body mass index and time to pregnancy among couples pregnant within a year: A China cohort study

**DOI:** 10.1371/journal.pone.0231751

**Published:** 2020-04-23

**Authors:** Yuhang Fang, Jun Liu, Yanyan Mao, Yang He, Min Li, Liu Yang, Qianxi Zhu, Qi Tong, Weijin Zhou

**Affiliations:** 1 School of Public Health, Fudan University, Shanghai, China; 2 NHC Key Lab. of Reproduction Regulation, Shanghai Institute of Planned Parenthood Research, Fudan University, Shanghai, China; 3 NHC Key Laboratory of Birth Defects and Reproductive Health (Chongqing Population and Family Planning Science and Technology Research Institute), Chongqing, China; University of Mississippi Medical Center, UNITED STATES

## Abstract

**Background:**

Extreme pre-pregnancy body mass index (BMI) values have been associated with reduced fecundability and prolonged time to pregnancy in previous studies. However, the effect in fertile couples is unclear.

**Objectives:**

This study aimed to evaluate the association between pre-pregnancy BMI and fecundability, measured as time to pregnancy (TTP), among couples that achieved pregnancy within 1 year.

**Methods:**

This was a retrospective cohort study of 50,927 couples wishing to conceive, enrolled in the National Free Preconception Health Examination Project (NFPHEP) in Chongqing, China, during 2012–2016. Participants’ weight and height were measured by NFPHEP-trained preconception guidance physicians. TTP measured in months was used to determine subfecundity (TTP >6 months). The strength of association between BMI and TTP/subfecundity was measured with fecundability odds ratios (FOR)/odds ratios (OR) and their corresponding 95% confidence intervals (CI), calculated with Cox and logistic regression analysis. We used restricted cubic spline regression (RCS) to test the observed FOR trends.

**Results:**

Compared to women with normal BMI, women with pre-pregnancy overweight/obesity had longer TTP (FOR = 0.96, 95% CI: 0.94–0.99) and increased risk of subfecundity (OR = 1.08, 95% CI: 1.00–1.17). There was no association between TTP and male BMI. RCS trends varied when data were stratified by male pre-pregnancy BMI, with the greatest change detected in pre-pregnancy underweight men.

**Conclusions:**

Pre-pregnancy overweight/obesity was associated with longer TTP and subfecundity among women who became pregnant within 1 year; this effect was likely mediated by their partners’ pre-pregnancy BMI. These findings indicate that BMI could affect fecundability, independently of affecting the risk of sterility. Advice on weight management and maintaining healthy weight should be included in couples’ preconception guidance.

## Introduction

Obesity affects 13% of the world population and 12.4% of adults in China [1, 2]. Previous research has suggested that obesity might damage couples’ reproductive capacity through several biological processes [3–6], such as hypogonadism and abnormal germ cell production. Concurrently, underweight could also reduce fecundability through increased secretion of FSH, secondary amenorrhea [7], and shortened luteal phase [8] as the prevalence of underweight were 9.7% and 7.8% for worldwide and Chinese women [9, 10].

Epidemiologists have used body mass index (BMI) [11] and time to pregnancy (TTP) [12] to assess the relationship between extreme body weight and fecundability at a population level. Some previous studies have suggested that lower fecundability, reflected in prolonged TTP, was associated with higher BMI or higher body fat percentage [11, 13–24], but these findings are inconsistent [25, 26]. Meanwhile, few previous studies have found that underweight was associated with decreased fecundability [17, 22, 23]. Methodologically, most previous studies have focused on an only one of the prospective parents with a follow-up period longer than 12 months [11, 14–19, 21–24, 26–28]. However, no previous study has evaluated the relationship between BMI and TTP in fertile populations (TTP ≤ 12 months). Moreover, previous studies have relied on participant self-reported data on TTP and BMI [15, 16, 19, 20, 22, 25]. Finally, previous studies’ participants tended to be women of reproductive age selected as part of a birth cohort [20, 25] or an occupational cohort [15, 16, 28].

This study aimed to clarify whether prospective parents’ extreme BMI values (obesity and underweight) affect TTP and increase the risk of subfecundity (TTP >6 months) in women who became pregnant within 12 months since beginning their efforts to conceive. To this end, we evaluated the association between pre-pregnancy BMI and fecundability (measured with TTP) among couples who conceived within 12 months of enrollment at the Chongqing’s pre-conception physical check-up service.

## Materials and methods

### Study population

This was a retrospective cohort study of women aged 20–49 and their spouses aged 22–60 years, enrolled in the National Free Preconception Health Examination Project (NFPHEP) in Chongqing. The project was launched by the Chinese National Health and Family Planning Commission and Ministry of Finance in 2010 to improve maternal and infant health [29, 30]. All NFPHEP participants received free pre-pregnancy health education, medical check-ups, counseling, and relevant follow-up services from trained staff.

### Ethical approval

Written informed consent was obtained from all couples. All data were kept strictly confidential. Institutional review board approvals were obtained from the Chongqing Population and Family Planning Science and Technology Research Institute (approved date: 2017–04).

### Data collection: Exposure, outcome and covariates

Data were extracted from NFPHEP records, collected during recruitment and follow-up visits, including demographic characteristics, disease and medication history, reproductive history, family history, lifestyle, environmental exposure, psychosocial stress, physical examination, and laboratory tests. Follow-up assessments were conducted over the phone every 3 months for up to 12 months after the preconception health examination. Urine pregnancy tests and type B ultrasound tests were conducted to confirm conception in women who self-reported pregnancy. NFPHEP follow-up form completed with women who were clinically diagnosed as pregnant.

Pre-pregnancy BMI was the exposure of interest, defined as body weight (kg) divided by height-squared (m^2^). Participants’ height and weight were measured by NFPHEP-trained preconception guidance physicians and recorded after the physical examination. The BMI cut-off points were based on the guidelines of the Working Group on Obesity in China (WGOC) [31], defined as follows: underweight ([UW] <18.5 kg/m^2^), normal weight ([NW] 18.5–23.9 kg/m^2^), overweight ([OW] 24–27.9 kg/m^2^), and obesity ([OB] ≥28 kg/m^2^).

In this study, the primary outcome of interest was time to pregnancy (TTP), used as a measure of fecundability, which is considered more objective and practical compared to biological indicators [32]. TTP was measured in months [33] and defined as the interval between the date of enrollment and the last menstrual period (LMP), as provided on the NFPHEP follow-up form. A follow-up month was defined as 30 days, the last 331–365 days was calculated as the 12th follow-up month. Fecundability was defined as the average probability of conception in each given month. The secondary outcome of interest in the present study was subfecundity (TTP >6 months) [34].

The following variables as potential confounders in this study were identified through reviewing previous relevant research literature [17, 35, 36]: age (<25, 25–29, 30–34, ≥35 years), type of household (urban, rural), education (≤primary, junior, senior, ≥college), cigarette exposure (no, yes), alcohol consumption (no, sometimes, often), stress (no, yes), age of menarche, menstrual cycle regularity (regular, irregular), gravidity (0, ≥1), parity (0, ≥1), spontaneous abortion (0, ≥1), induced abortion (0, ≥1), details of which were extracted from the NFPHEP database. Psychosocial stress was defined based on answers to the following questions: “Do you feel the pressure of life/work? Has it been tense with friends, relatives, and colleagues? Do you feel economic pressure?” The answers were: “none,” “seldom,” “a little bit,” “a lot,” and “a great deal.” All answers to these questions were indicative of psychological stress, except “none” to all three, which indicated absence of psychological stress.

### Statistical analysis

Women with OW and OB were combined into a single group due to the fewer number of obese women. Proportions and chi-square tests were used to describe and compare discrete variables between the UW, NW, and OW/OB groups. Means and standard deviations (SD), and one-way analysis of variance (ANOVA) were used to describe and examine the continuous variables. Multivariate Cox regression analysis (failure event = conceive) were used to estimate the fecundability odds ratios (FOR) and the corresponding 95% confidential intervals (CI) for the underweight or overweight/obesity group compared to the normal weight group in men and women. FOR >1 indicated shorter TTP; FOR <1 indicated longer TTP [13]. To confirm Cox regression analysis results for the categorical BMI analysis, we used restricted cubic splines (RCS) to test the observed FOR trends. RCS were fitted with 3 knots (18.5, 20.7, 24.0) of female BMI, with the BMI reference value of 22 kg/m^2^. The spline curves for the women’s pre-pregnancy BMI and FOR were further stratified according to their partners’ pre-pregnancy BMI. Logistic regression analysis was used to calculate odds ratio (OR) with 95% CI for the association between subfecundity and BMI by comparing outcomes of the UW or OW/OB group to the outcomes of the NW group. All statistical analyses were conducted with SAS software (version 9.4, SAS Institute, Cary, NC, USA), with a two-sided p-value <0.05 was considered statistically significant.

## Results

There were 81,916 pregnant couples enrolled in the NFPHEP at the Chongqing Municipality during 2012–2016. Of these, 24,051 were pregnant before enrollment, and 6 had a follow-up date of more than 1 year, while 1,930 couples had missing age data or were of age that was outside of the eligibility range set for this study. A total of 5,002 couples were excluded due to missing information on pre-pregnancy BMI or one of the prospective parents having an extreme pre-pregnancy BMI value (women <14.5 or >35.5 kg/m^2^, men <15.5 or >38.0 kg/m^2^). Finally, 50,927 couples were included in this study.

The average pre-pregnancy BMI (kg/m^2^) for women and men was 21.0 (SD: 2.6) and 22.9 (SD: 3.1), respectively. Distribution of pre-pregnancy UW, NW, OW, and OB was 15.0%, 72.2%, 11.0%, and 1.8%, respectively, among women; and 5.2%, 61.0%, 27.5%, and 6.3%, respectively, among men. Demographic characteristics of the included couples, stratified by BMI, are summarized in Table 1. Women and men in the pre-pregnancy OW/OB group were more likely to be older, more often exposed to passive smoking, and more likely ready for pregnancy than women and men in the pre-pregnancy UW or NW groups. Men in the pre-pregnancy OW/OB group were more likely to be based within urban areas, have achieved higher education level, and have lower exposure to smoking, and higher psychosocial stress, which was in contrast to women in this group, for whom these relationships were reversed. Men with pre-pregnancy OW/OB consumed more alcohol. Women with pre-pregnancy OW/OB were more likely to be multiparas, have an irregular menstrual cycle, and experience spontaneous or induced abortion (Table 2).

**Table 1:** Demographic characteristics of couples included in this study, stratified by BMI categories.

Variables	Male BMI n(%)/ $\bar{x}\pm s$				Female BMI n(%)/ $\bar{x}\pm s$			
	<18.5 (n = 2631)	18.5–23.9 (n = 31048)	≥24 (n = 17248)	P	<18.5 (n = 7639)	18.5–23.9 (n = 36736)	≥24 (n = 6552)	P
**Age, year**								
(continuous)	26.36±3.64	27.72±4.52	28.90±4.76	<0.0001	24.80±3.18	25.78±4.04	27.21±4.97	<0.0001
<25	896(34.1)	7594(24.5)	2780(16.1)	<0.0001	3691(48.3)	15155(41.3)	2149(32.8)	<0.0001
25~	1323(50.3)	15197(48.9)	8133(47.1)		3424(44.8)	16036(43.6)	2629(40.1)	
30~	319(12.1)	5536(17.8)	4150(24.1)		455(6.0)	4164(11.3)	1160(17.7)	
35~	93(3.5)	2721(8.8)	2185(12.7)		69(0.9)	1381(3.8)	614(9.4)	
**Ethnicity**								
Han	2478(95.4)	28761(93.9)	16101(94.5)	0.0017	7155(94.9)	34041(93.9)	5973(92.2)	<0.0001
Non-Han	120(4.6)	1855(6.1)	941(5.5)		385(5.1)	2196(6.1)	504(7.8)	
Missing	33	432	206		99	499	75	
**Type of household**								
Ag	1874(71.2)	21225(68.5)	9967(57.8)	<0.0001	5031(65.9)	25111(68.4)	4600(70.2)	<0.0001
Non-Ag	757(28.8)	9793(31.5)	7281(42.2)		2608(34.1)	11625(31.6)	1952(29.8)	
**Education**								
≤ Primary	97(3.8)	1119(3.7)	517(3.1)	<0.0001	156(2.1)	1222(3.5)	477(7.5)	<0.0001
Junior	1051(41.5)	12487(41.8)	6013(36.1)		2323(31.9)	14267(40.4)	3096(48.6)	
Senior	792(31.2)	7347(24.6)	3672(22.1)		1956(26.9)	8240(23.4)	1303(20.5)	
≥ College	596(23.5)	8945(29.9)	6434(38.7)		2841(39.1)	11534(32.7)	1488(23.4)	
Missing	95	1150	612		363	1473	188	
**Occupation**								
Peasant	935(36.6)	11844(39.3)	5250(31.54)	<0.0001	2358(32.0)	13995(39.3)	2729(42.6)	<0.0001
Worker	548(21.4)	5514(18.3)	2957(17.7)		796(10.8)	3529(9.9)	742(11.6)	
Service industry	290(11.3)	3084(10.2)	1730(10.4)		903(12.2)	4143(11.6)	635(9.9)	
Business	149(5.8)	1491(5.0)	1055(6.3)		320(4.3)	1304(3.7)	210(3.3)	
Housework	13(0.5)	137(0.5)	62(0.4)		428(5.8)	1956(5.5)	546(8.5)	
Teachers/Civil servants	274(10.7)	4799(15.9)	3617(21.7)		1696(23.0)	7147(20.1)	892(13.9)	
Others	349(13.7)	3263(10.8)	2020(12.1)		878(11.9)	3529(9.9)	657(10.2)	
Missing	73	916	557		265	1136	145	
**Smoking**								
No	1349(51.3)	18935(61.1)	10638(61.8)	<0.0001	7567(99.3)	36412(99.3)	6461(98.8)	<0.0001
Yes	1279(48.7)	12071(38.9)	6583(38.2)		50(0.7)	244(0.7)	76(1.2)	
Missing	3	42	27		22	80	15	
**Passive smoking**								
No	1947(74.2)	23177(74.9)	12725(74.0)	0.0001	6262(82.1)	30890(84.2)	5538(84.7)	<0.0001
Sometimes	632(24.1)	7317(23.6)	4129(24.0)		1227(16.1)	5174(14.1)	846(12.9)	
Often	44(1.7)	451(1.5)	348(2.0)		137(1.8)	604(1.7)	153(2.3)	
Missing	8	103	46		13	68	15	
**Alcohol consumption**								
No	1566(59.6)	18313(59.1)	9529(55.3)	<0.001	7057(92.6)	34193(93.3)	6125(93.6)	0.0501
Sometimes	1020(38.8)	12139(39.2)	7326(42.5)		561(7.3)	2428(6.6)	412(6.3)	
Often	40(1.5)	534(1.7)	369(2.2)		1(0.1)	17(0.1)	4(0.1)	
Missing	5	62	24		20	98	11	
**Stress**								
Yes	509(19.4)	6309(20.4)	3733(21.7)	0.0006	1135(14.9)	4991(13.6)	747(11.4)	<0.0001
No	2113(80.6)	24674(79.6)	13484(78.3)		6483(85.1)	31685(86.4)	5792(88.6)	
missing	9	65	31		21	60	13	
**Ready for pregnancy**								
No	29(1.1)	355(1.2)	145(0.8)	0.0066	144(1.9)	584(1.6)	102(1.6)	0.1586
Yes	2596(98.9)	30648(98.8)	17089(99.2)		7488(98.1)	36113(98.4)	6442(98.4)	
missing	6	45	14		7	39	8	

**Table 2:** Gynecological history of the women included in this study, stratified by BMI categories.

Variables	Female Body Mass Index n(%)/ $\bar{x}\pm s$			
	<18.5 (n = 7639)	18.5–23.9 (n = 36736)	≥24 (n = 6552)	P
**Age of menarche, year**	13.37±1.13	13.29±1.09	13.22±1.14	<0.0001
Missing	14	82	16	
**Menstrual cycle regularity**				
Regular	7265(95.3)	35114(96.0)	6172(94.5)	<0.0001
Irregular	354(4.7)	1467(4.0)	356(5.5)	
Missing	20	155	24	
**Gravidity**				
0	3908(51.2)	16466(44.8)	1842(28.1)	<0.0001
≥1	3724(48.8)	20245(55.2)	4708(71.9)	
Missing	7	25	2	
**Parity**				
0	6159(80.7)	25166(68.5)	3179(48.5)	<0.0001
≥1	1473(19.3)	11545(31.5)	3371(51.5)	
Missing	7	25	2	
**Spontaneous abortion**				
0	7263(95.2)	34826(94.9)	6140(93.7)	0.0002
≥1	369(4.8)	1885(5.1)	410(6.3)	
Missing	7	25	2	
**Induced abortion**				
0	4823(63.2)	22723(61.9)	3418(52.2)	<0.0001
≥1	2809(36.8)	13988(38.1)	3132(47.8)	
Missing	7	25	2	

The total cumulative time to pregnancy for the included couples was 176,826 months, while 30,691 (60.0%) of the couples conceived in the first quarter following enrollment. The average TTP (months) for the UW, NW, and OW/OB groups was 3.50, 3.45, and 3.56, respectively, for women, and 3.46, 3.44, and 3.53, respectively, for men. Pre-pregnancy BMI was roughly associated with TTP among women and men; however, this association for men disappeared after adjusting for demographic characteristics. For women, pre-pregnancy OW/OB was associated with an increased risk of longer TTP (aFOR = 0.96, 95% CI: 0.94–0.99) compared to NW (Table 3). We reanalyzed our data, and using TTP measured in the number of cycles, and achieved similar results (S1 Table). Stratified by male BMI, female pre-pregnancy OW/OB was a risk factor for prolonged TTP (aFOR = 0.96, 95% CI: 0.92–1.00) when their partners were overweight or obese (S2 table).

**Table 3:** Association between pre-pregnancy BMI and TTP in men and women, Cox regression analysis.

Variables	Fecundability	cFOR(95%CI)	aFOR^a^(95%CI)	aFOR^b^(95%CI)
**Female BMI**				
<18.5	7639/26720	0.99(0.96–1.01)	0.99(0.96–1.01)	0.99(0.96–1.02)
18.5–23.9	36736/126808	Ref.	Ref.	Ref.
≥24	6552/23298	0.97(0.94–0.99)	0.96(0.93–0.99)	0.96(0.94–0.99)
**Male BMI**				
<18.5	2631/9112	0.99(0.95–1.03)	0.98(0.94–1.02)	0.98(0.94–1.02)
18.5–23.9	31048/106814	Ref.	Ref.	Ref.
≥24	17248/60900	0.97(0.95–0.99)	1.00(0.98–1.02)	1.01(0.98–1.02)

a: Female adjusted for women’s age (categorical), type of household, education, smoking, alcohol consumption, psychosocial pressure and ready for pregnancy; Male adjusted for men’s’ age (categorical), type of household, education, smoking, alcohol consumption, psychosocial pressure and ready for pregnancy.

b: adjusted for all variables in “a” plus cycle regularity, age of menarche, gravidity, spontaneous abortion and induced abortion.

**Fig 1:**
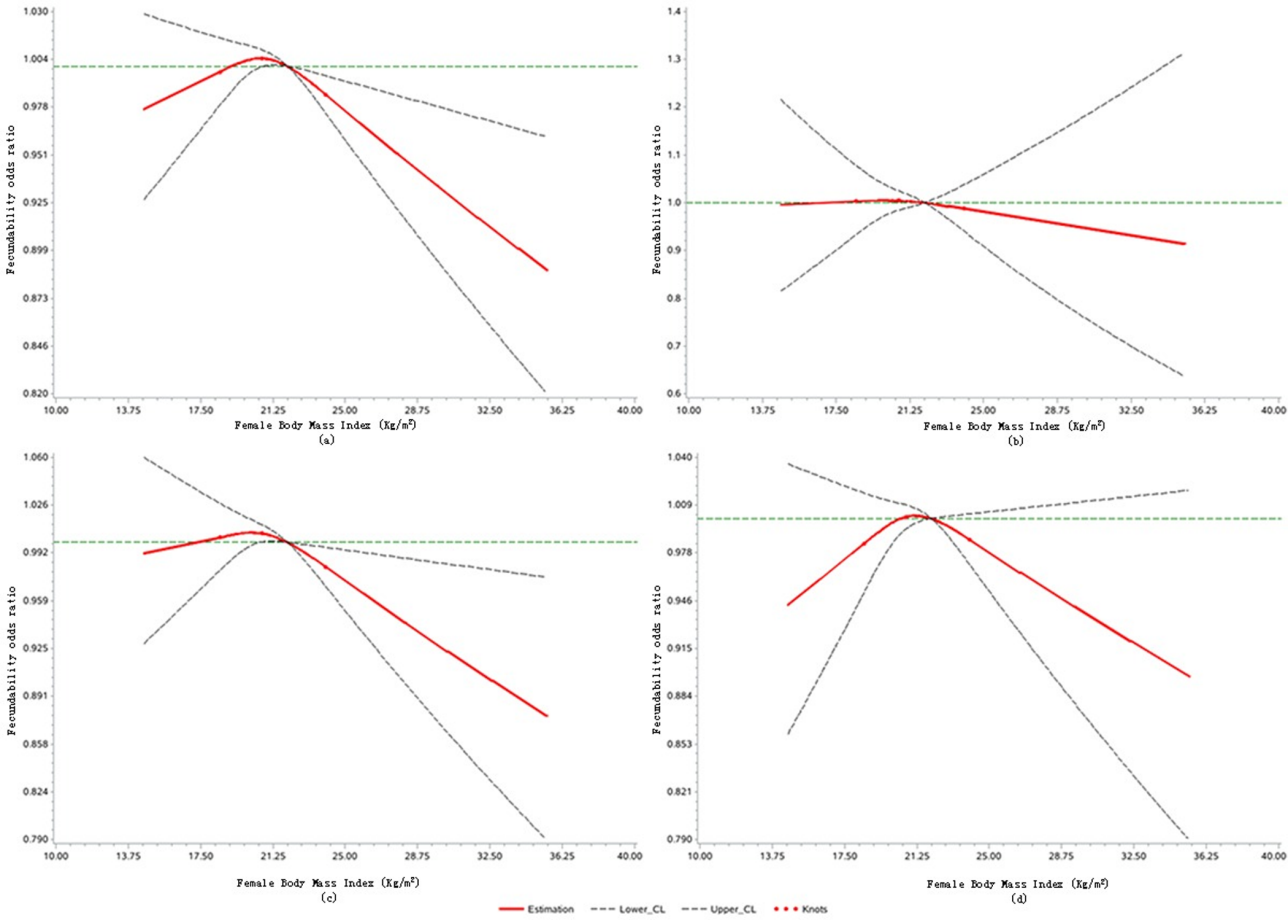
Association between female BMI and fecundability odds ratio, fitted by restricted cubic splines. (a) All pregnant women; (b) Pregnant women with UW spouse; (c) Pregnant women with NW spouse; (d) Pregnant women with OW/OB spouse. Knots are located at 18.5, 20.7, and 24.0 kg/m^2^. Reference level for FOR = 22 kg/m^2^. Reference line is Y = 1. The curves are adjusted for female age (categorical), type of household, education, smoking, alcohol consumption, psychosocial pressure, ready for pregnancy, cycle regularity, and age of menarche, gravidity, and spontaneous and induced abortion.

Restricted cubic splines analysis has shown that the association between female BMI and FOR was non-linear (p = 0.029). Compared with the reference group with BMI = 22 kg/m^2^, women with a BMI between 20.8 and 21.9 had a higher probability of conception; fecundability decreased as BMI increased since 22 kg/m (Fig 1A). Compared to the counterparts with the normal BMI, women with a lower BMI or a higher BMI seem to have a lower fecundability when their partner was in the OW/OB group, but the 95% CI were too wide (Fig 1D).

In a logistic regression analysis, female pre-pregnancy OW/OB (relative to NW) was associated with increased risk of subfecundity (OR: 1.08, 95% CI: 1.00–1.17). No association was observed between subfecundity and both partners’ pre-pregnancy UW, or male pre-pregnancy OW/OB (Table 4).

**Table 4:** Association between pre-pregnancy BMI and subfecundity in men and women, logistic regression analysis.

Variables	N	Case	P*	cOR(95%CI)	aOR^a^(95%CI)	aOR^b^(95%CI)
**Female BMI**						
<18.5	7639	1068	0.09	1.04(0.97–1.12)	1.03(0.96–1.11)	1.03(0.95–1.11)
18.5–23.9	36736	4948		Ref.	Ref.	Ref.
≥24	6552	943		1.08(1.01–1.16)	1.09(1.01, 1.18)	1.08(1.00–1.17)
**Male BMI**						
<18.5	2631	362	0.42	1.02(0.91–1.15)	1.07(0.95–1.20)	1.06(0.94–1.19)
18.5–23.9	31048	4194		Ref.	Ref.	Ref.
≥24	17248	2403		1.04(0.98–1.09)	0.97(0.92–1.03)	0.97(0.92–1.03)

* chi-square test.

a: Female adjusted for women’s age (categorical), type of household, education, smoking, alcohol consumption, psychosocial pressure and ready for pregnancy; Male adjusted for men’s’ age (categorical), type of household, education, smoking, alcohol consumption, psychosocial pressure and ready for pregnancy.

b: adjusted for all variables in “a” plus cycle regularity, age of menarche, gravidity, spontaneous abortion and induced abortion.

## Discussion

This large pre-conception cohort study of 50,927 fertile Chinese couples examined the association between pre-pregnancy BMI and fecundability, measured by TTP. We found reduced fecundability and an increased risk of subfecundity among women with pre-pregnancy OW/OB compared ones with normal BMI. In fact, fecundability decreased as female BMI increased. Female pre-pregnancy UW and male pre-pregnancy BMI were not associated with diminished fecundability. However, the association between female pre-pregnancy OW/OB and prolonged TTP varied when stratified by partners’ BMI.

To the best of our knowledge, this is the first study to evaluate the association between female pre-pregnancy BMI and TTP among fertile couples who achieved conception within 12 months of enrollment. Our findings are consistent with the results of the previous studies, which have shown that woman’s fecundability declines with the increase of BMI [11, 14–16, 21]. A previous study based in Singapore has suggested that East Asian populations might have a lower BMI threshold for prolonged TTP [11]. A previous birth cohort study [25] of fertile women has reported point estimates of FOR similar to the estimates reported in the present study; however, the previously reported estimates were not statistically significant, likely due to a small sample size of 1924 couples and reference group with 20−25 kg/m^2^ of BMI. Two studies of internet-based pre-conception cohorts have demonstrated that fecundability decreases with increasing BMI [14, 19]; however, these findings were statistically significant only for North American participants who were very obese (≥40 kg/m^2^) [14], so did African-American women [16]. These American study divided BMI into overweight and various levels of obesity, meanwhile, we combined overweight and obesity into one group for comparison. FOR decreased with the increase of BMI, so the effect of combined group was stronger than the overweight group alone. In contrast to previous studies that included cases of infertility during follow-up [11, 13–15], our study included only couples that were able to conceive within 12 months.

The association between female pre-pregnancy OW/OB and subfecundity reported in the present study was supported by previous studies [20, 22, 23]. However, one antenatal clinics-based study found the statistical association between pre-pregnancy underweight and subfecundity [22]. In addition, cut-off points for subfecundity used in these studies were inconsistent, set at 9.5 [23] or 12 months [20, 22].

In men, a few previous studies have found that high BMI [20, 27, 28] was associated with decreased fecundability, and no recent studies have reported similar findings [13, 25]. However, more than half of Norwegian men were OW/OB [27] and used BMI reference group between 20.0 and 22.5 kg/m^2^, so the study was more prone to statistical significance. In Agricultural Health Study, when OW/OB and occupational exposure were highly correlated, the results might be affected by pesticides [28]. And these studies focused on infertility [20, 27, 28] rather than changes to fecundability in a fertile population. Although in the present study there was no impact of male BMI on fecundability, FORs for women with OW/OB varied when stratified by male BMI, suggesting that the effect of female BMI on TTP might be modified by their partners’ BMI. Only two previous studies have reported on the impact of both prospective parents’ BMI on TTP [13, 20]. A Danish study has found that women with obesity partnered with underweight men were most likely to experience extended TTP, with a highest OR of 3.79 (95% CI: 1.48–9.74) [20]. However, the Longitudinal Investigation of Fertility and the Environment (LIFE) Study, based in Michigan and Texas in the United States, has shown reduced fecundability among couples with obesity (FOR = 0.41, 95% CI: 0.17, 0.98) [13]. Both studies likely included couples that took >12 months to conceive. In our study, in a more fertile population (TTP ≤ 12 months), we also observed slightly lower fecundability among women whose partners were underweight.

There are several possible underlying mechanisms for the association between BMI and TTP. For example, obesity could cause ovulatory dysfunction, sex hormone disorders, and metabolic syndrome by increasing secretion of estrogen and leptin, and decreasing levels of gonadotropins and progestin [37, 38]. Meanwhile, obesity might also be associated with injured endometrial receptivity [39], implantation [40], and organic diseases such as polycystic ovarian syndrome (PCOS) [41]. Finally, inflammation [42] and fatty acid toxicity [43], which occurs more frequently among obese women, could damage the eggs and impair functioning of the reproductive organs. Extremely high BMI could affect semen quality [44]; however, it is plausible that it would not affect the likelihood of conception given a large number of sperm cells; this observation might partially explain the absence of association between men’s pre-pregnancy BMI and TTP. Couples’ BMI generally increases with age; in men, being underweight might indicate malnutrition or subclinical physiological diseases, which might affect sperm quality, and thus TTP [44, 45].

This study has several strengths. First, this was a large population-based retrospective cohort study based on the NFPHEP. All the BMI data were provided before pregnancy. Although the participants of this study were residents of 39 counties across the whole of the Chongqing Municipality, measuring instruments and protocols were uniform across the project sites, ensuring the consistency of measurements. Second, we estimated the effect of men’s and women’s BMI on TTP and evaluated the modifying effect of partners’ BMI. Finally, studies with a sample composition restricted to fertile individuals, assessing the relationship between BMI and fecundability, based in countries with well-established family planning systems, such as China, could provide evidence relevant to long-term and multi-children family planning.

The major limitation of the present study is that obesity was only ascertained through BMI, as data on body composition and shape, such as waist-to-hip ratio, skinfold thickness, and total body fat percentage were not available. The estimated nature effect of the prospective parents’ BMI on TTP was likely underestimated in the present study, as couples who had not reported pregnancy within 1 year of enrollment (TTP>12 months) were excluded. In addition, our study estimates did not account for unknown and unmeasured residual confounding, including intercourse frequency, physical activity, and weight changes [14, 15]. In our study, we lacked information of assisted reproductive technology (ART), so the bias by ART in this study could not be ruled out completely. Finally, as we had no data on pregnancy attempts prior to project enrollment, we were not able to control for them in our analysis. Future research should account for body shape and composition, timescales of pregnancy attempts, and intercourse frequency for more accurate estimates.

In conclusion, fecundability impairment link to pre-pregnancy OW/OB might be present in fertile women, as manifested by longer time to pregnancy and increased risk of subfecundity. Maternal pre-pregnancy BMI, which might interact with partners’ BMI, plays a role in determining TTP during the first year of pregnancy attempts. The weight intervention prior to conception could be considered in a general population of trying to conceive.

## Supporting information

S1 Table Association between pre-pregnancy BMI and TTP, measured by cycle. (DOCX)

S2 Table Association between maternal pre-pregnancy BMI and TTP, stratified by paternal BMI. (DOCX)

S1 File The data set used for analysis in this study. (CSV)
