# Supplementary material for: Pre-pregnancy body mass index and time to pregnancy among couples pregnant within a year: A China cohort study
Source: PLoS One. 2020 Apr 23;15(4):e0231751. doi: 10.1371/journal.pone.0231751 (PMC7179844; doi:10.1371/journal.pone.0231751)
Supplement: S1 Table — (DOCX) [file pone.0231751.s001.docx]

**S1 table. Association between pre-pregnancy BMI and TTP, measured by cycle**

| **Variables** | **Fecundability** | **cFOR(95%CI)** | **aFOR^a^(95%CI)** | **aFOR^b^(95%CI)** |
| --- | --- | --- | --- | --- |
| **Female BMI** |  |  |  |  |
| <18.5 | 6047/21299 | 1.00(0.97–1.02) | 1.00(0.97–1.03) | 1.00(0.97–1.03) |
| 18.5-23.9 | 28718/100625 | Ref. | Ref. | Ref. |
| ≥24 | 5056/18147 | 0.97(0.95–1.00) | 0.97(0.94–1.00) | 0.97(0.94–1.00) |
|  |  |  |  |  |
| **Male BMI** |  |  |  |  |
| <18.5 | 2080/7283 | 0.99(0.95–1.04) | 0.99(0.94–1.03) | 0.99(0.94–1.03) |
| 18.5-23.9 | 24291/84580 | Ref. | Ref. | Ref. |
| ≥24 | 13450/48208 | 0.97(0.95–0.99) | 1.00(0.98–1.02) | 1.00(0.98–1.02) |

N=39821

a: Female adjusted for women’s age (categorical), type of household, education, smoking, alcohol consumption, psychosocial pressure and ready for pregnancy; Male adjusted for men’s’ age (categorical), type of household, education, smoking, alcohol consumption, psychosocial pressure and ready for pregnancy.

b: adjusted for all variables in “a” plus age of menarche, gravidity, spontaneous abortion and induced abortion.
