# Supplementary material for: Pre-pregnancy body mass index and time to pregnancy among couples pregnant within a year: A China cohort study
Source: PLoS One. 2020 Apr 23;15(4):e0231751. doi: 10.1371/journal.pone.0231751 (PMC7179844; doi:10.1371/journal.pone.0231751)
Supplement: S2 Table — (DOCX) [file pone.0231751.s002.docx]

**S2 Table. Association between maternal pre-pregnancy BMI and TTP, measured by month, stratified by paternal BMI**

| **Female BMI** |  | **Fecundability** | **cFOR(95%CI)** | **aFOR(95%CI)** |
| --- | --- | --- | --- | --- |
| Male BMI <18.5 | | | | |
| <18.5 |  | 544/1879 | 0.98(0.89–1.08) | 1.00(0.90–1.10) |
| 18.5-23.9 |  | 1806/6190 | Ref. | Ref. |
| ≥24 |  | 281/1043 | 0.89(0.78–1.01) | 0.90(0.79–1.02) |
|  |  |  |  |  |
| Male BMI 18.5-23.9 | | | | |
| <18.5 |  | 4794/16631 | 0.99(0.96–1.02) | 0.99(0.96–1.03) |
| 18.5-23.9 |  | 22595/77370 | Ref. | Ref. |
| ≥24 |  | 3659/12813 | 0.98(0.94–1.01) | 0.97(0.94–1.01) |
|  |  |  |  |  |
| Male BMI ≥24 | | | | |
| <18.5 |  | 2301/8210 | 0.98(0.94–1.03) | 0.98(0.93–1.02) |
| 18.5-23.9 |  | 12335/43248 | Ref. | Ref. |
| ≥24 |  | 2612/9442 | 0.97(0.93–1.01) | 0.96(0.92–1.00) |

Adjusted for age (categorical), type of household, education, smoking, alcohol consumption, psychosocial pressure, ready for pregnancy, cycle regularity, and age of menarche, gravidity, spontaneous abortion and induced abortion.
